# Early-career researchers’ views on ethical dimensions of patient engagement in research

**DOI:** 10.1186/s12910-018-0260-y

**Published:** 2018-03-07

**Authors:** Jean-Christophe Bélisle-Pipon, Geneviève Rouleau, Stanislav Birko

**Affiliations:** 1 Petrie-Flom Center, Harvard Law School, 23 Everett St., Room 324, Cambridge, MA 02138 USA; 2 Health Law Institute, Dalhousie University, Halifax, NS Canada; 3 Faculty of Nursing, Laval University, Quebec City, QC Canada; 4 University of Montreal Hospital Research Centre, Montreal, QC Canada; 5 Bioethics Program, University of Montreal School of Public Health, Montreal, QC Canada; 6 University of Montreal Public Health Research Institute, Montreal, QC Canada

**Keywords:** Patient engagement, Patient-oriented research, Patient-centered outcomes, Ethical aspects, Tokenism, Authorship, Ethical preparedness

## Abstract

**Background:**

Increasing attention and efforts are being put towards engaging patients in health research, and some have even argued that patient engagement in research (PER) is an ethical imperative. Yet there is relatively little empirical data on ethical issues associated with PER.

**Methods:**

A three-round Delphi survey was conducted with a panel of early-career researchers (ECRs) involved in PER. One of the objectives was to examine the ethical dimensions of PER as well as ECRs’ self-perceived level of preparedness to conduct PER ethically. The study was conducted among awardees of the Québec SPOR-SUPPORT Unit in Canada, who represent the next generation of researchers involved in PER. Many themes were addressed throughout the study, such as definition, values, patients’ roles, expected characteristics of patients, and anticipated challenges (including ethical issues). Open-ended questions were used, and all quantitative data were collected through statements using 7-point Likert scales.

**Results:**

Between April and November 2016, 25 ECRs were invited to participate; 18 completed both the first and second rounds, and 16 completed the third round. Panelists consisted of nine women and seven men with various backgrounds (general practitioners and postgraduate students). The majority were between 25 and 44 years old. Panelists’ responses showed PER raises important ethical issues: 1) professionalization of patients involved in research (with risks of patients becoming less representative); 2) adequate remuneration of patients; 3) fair recognition of patients’ experiential knowledge; and 4) tokenism (engaging patients only for symbolic appeal). While the panelists felt moderately prepared to confront these ethical issues, they reported being uncomfortable applying for an ethics certificate for a PER project.

**Conclusion:**

If PER is an ethical imperative, it is vital to establish clear ethical standards and to train and support the PER community to identify and resolve ethical issues. Despite their overall readiness to conduct PER, panelists did not feel adequately prepared to address many of these issues. It is not easy for ECRs to reconcile ethical desiderata and logistical imperatives. Additional research should focus on supporting the responsible conduct of PER, which, if not done, can undermine the credibility and feasibility of the entire PER enterprise.

## Background

While health research has historically been conducted mainly *on* patients, there is a growing expectation that research should be conducted *with* patients. Engaging patients is presented as a means of transcending the traditional paternalistic view of patients as passive subjects lacking the ‘proper’ expertise and knowledge to guide research. Stephens and Staniszewska refer to “a fundamental paradigm shift in health and social care research, away from paternalism towards partnership” [1]. However, there exists an “epistemological dissonance” or “know-do gap” [2] between researchers’ views of the potential positive consequences of patient engagement in research (PER) and their actual practices. Although PER is not yet widespread, it has been conceptualized as research oriented towards two common and complementary goals: integrating patients’ experiential knowledge and fostering patient empowerment [3, 4].

In the literature, there is a tendency to consider PER as an ethical imperative (i.e., that it *should* be conducted based on ethical grounds) [3, 5, 6]. This view is predicated on the principle that research must serve those on whom and for whom it is conducted. Domecq Garces et al. argue that PER is morally compelling, since patients are the “ultimate user of research evidence” [6], while Solomon et al. note that PER enables research to better meet community priorities and helps build “trustworthy research that communities can believe in” [5]. PER is ethically appropriate, according to Hardavella et al., because it lays the ground for the necessary partnership―based on core values such as openness, transparency, and public accountability―between patients and researchers that benefits both [3]. Shippee et al. consider that PER can be justified not only on deontological grounds, since there is “a moral/ethical drive to empower lay participants in an otherwise expert-dominated endeavor and ensure civically responsible research”, but also from a consequentialist standpoint, since PER can improve the effectiveness of research projects and resulting interventions [7].

Funding agencies such as the Patient-Centered Outcomes Research Institute (PCORI) in the US, the Canadian Institutes of Health Research (CIHR), and the UK’s National Institute of Health Research (NIHR) acknowledge that PER raises specific ethical challenges that must be considered in research ethics reviews [8–10], but their strategies and reports do not discuss how researchers should be empowered to tackle the ethical issues raised by PER, nor the ethical imperative to engage patients in research.

PER is an emerging field whose definitional and operational frameworks are still being debated [11, 12]. The field remains empirically uncharted with regard to research integrity (i.e., responsible conduct of PER projects) and ethics (i.e., applying ethical standards for respecting and protecting human participants). Early-career researchers (ECRs) represent the next generation who, unlike previous ones, will have to contend with PER as a dimension of health research throughout their careers. ECRs’ development of PER-related skills, acuity, and expertise―which senior researchers will have had to acquire during their careers rather than in their training―will help foster the new research paradigm of meaningful patient engagement. ECRs are often considered to be in a constrained and precarious transitional phase from dependent to independent research [13, 14], for which the factors facilitating career development are not yet very well understood [15, 16].

To support them in this transition to becoming fully independent researchers conducting PER projects, we argue it is important that the research community understand how ECRs perceive: 1) the most compelling ethical issues they are likely to encounter; 2) their level of preparedness to deal with ethical challenges; and 3) what they need in order to engage patients responsibly in their projects. While absent in the literature, these elements are crucial to understand the scope of this emerging ethical imperative and to identify avenues of action and reflection to support the transition.

This paper presents the ethical aspects raised by participants in a broader Delphi survey whose primary aim was to examine how PER is being defined and circumscribed, identify its most pressing issues, and encourage the formulation of recommendations to support ECRs conducting PER. While the study was not initially intended to focus on the ethical dimensions of PER, these were considered important by participants in the first Delphi round and so became part of the analysis.

## Methods

Between April and November 2016, a Delphi survey was conducted with a panel of ECRs in the province of Quebec, Canada. Ethics approval was obtained through the University of Montreal Health Research Ethics Committee (#16–044-CERES-D) and an electronic informed consent was obtained from the participants. The Delphi methodology was chosen because it enables structured, confidential, and asynchronous group communication, thus avoiding the drawbacks of non-anonymous group communications [17, 18]. Three rounds were used: a ‘classic’ first round [19] of open-ended questions regarding panelists’ experience with PER and its definition, values, feasibility, and utility; a second round to deepen and clarify salient issues emerging from Round 1; and a third round consisting of four second-round questions that had not demonstrated consensus within the group and 14 new questions aimed at generating more precise recommendations.

Participants were drawn from the 25 awardees of the Quebec Strategy for Patient-Oriented Research and Support for People and Patient-Oriented Research and Trials (SPOR-SUPPORT) Unit in March 2016 [20]. Of the 25 invited to participate, 18 (72%) completed both the first and second rounds, and 16 of those (89%) completed the third round. This final group consisted of nine women and seven men. In terms of age, one participant was under 25 years, seven were between 25 and 34, seven were between 35 and 44, and one between 45 and 54. All 16 participants had bachelor’s degrees, 15 had master’s degrees, six were MDs, six were PhDs (two of whom were also MDs), and four had postdoctoral research experience. Twelve participants identified their main occupation as student, two as clinician, and two as dividing time between clinical work and teaching. Main self-identified areas of expertise were: medicine (5), public health (2), pharmacology (2), health sciences (2), chemistry (1), epidemiology (1), clinical research (1), nursing (1), and bioethics (1). Knowledge of PER was self-assessed as: “becoming familiar with PER” (4), “basic knowledge of PER” (6), “advanced knowledge of PER” (5), and “mastery of PER” (1).

Qualitative analysis was conducted using QDA Miner 4.1.27 and NVivo Version 11. Quantitative responses were analyzed using Excel and SPSS 22. All Likert scales consisted of seven points ranging from 1 (“completely disagree”) to 7 (“completely agree”).

## Results

After briefly considering the panelists’ conceptions of PER, this section will discuss the values specific to PER judged as most relevant, the ethical issues deemed most pressing, panelists’ self-perceived ethical preparedness for conducting PER, issues of authorship, and concerns over tokenism.

### What is PER?

Panelists agreed on a broad, three-pronged definition of PER that consisted of: 1) valuing, mobilizing, and legitimizing the experiential knowledge of patients living with a particular health condition; 2) conducting research that focuses on patients’ concerns, participation, and outcomes; and 3) integrating active partnership among a variety of actors (researchers, clinicians, decision-makers, institutions, patients, families, etc.). Panelists generally agreed that the most accurate terms for referring to patients engaged in research are *patient-partners in research* (PPRs) (7/16) and *patients engaged/involved in research* (5/16). Panelists considered PPRs to be full members of the research team (mean = 5.33), but were only moderately convinced that PPRs should be considered co-investigators (m = 4.66), instead attributing to them an advisory role (m = 5) in which they can voice opinions and participate in decision-making throughout the research process. Panelists concurred (m = 5.56) that it is realistic to expect that, from the start of a PER project, a research team should agree on the nature of the commitment of each actor involved in the research, including patients.

### Values

**Fig. 1:**
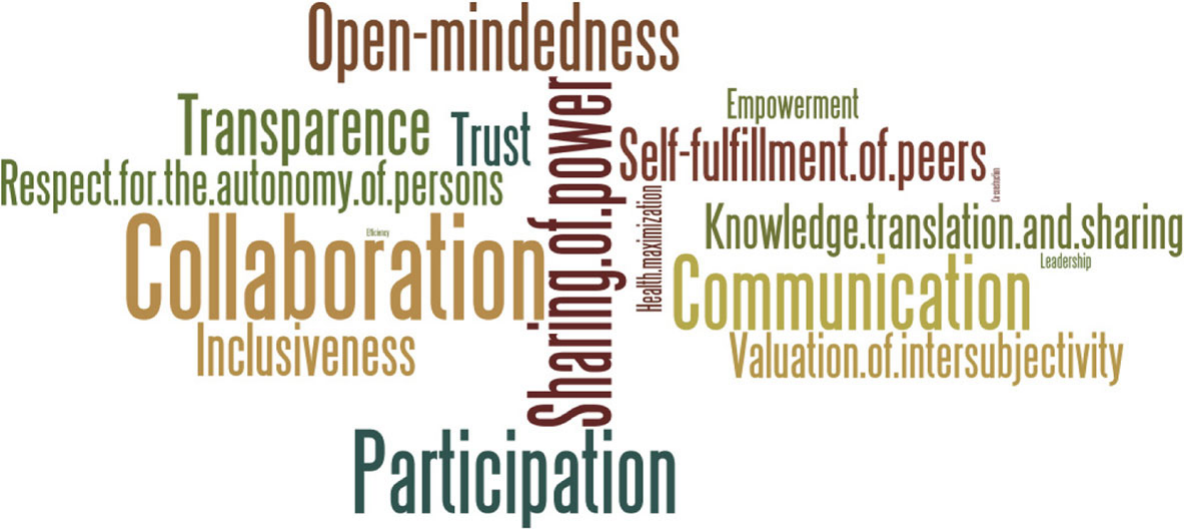
The values underlying PER as a word cloud, with size equal to ranking

The panelists identified values underlying research that are specifically relevant to PER. After scoping the field in Round 1, in subsequent rounds they narrowed the values to those they considered most important. Figure 1 presents the final aggregated results. The most important values were related either to the nature of the relationship (collaboration, participation, communication), the egalitarian aspect of PER (sharing of power), or the attitudes of the actors involved (open-mindedness, inclusiveness). While occupying a cardinal position in North American bioethics, respect for patients’ autonomy was ranked only 10 out of 19, tied with trust. Empowerment and health maximization were ranked 13 and 14, respectively. Efficacy (of research) and patients’ taking control were mentioned in Round 1, but never selected in subsequent rounds as important values in PER.

### Most pressing ethical issues

Using the process detailed above, panelists were asked to identify the ethical issues they considered most important. Table 1 presents the top 10 ethical issues they identified together with the profiles of panelists more likely to select each given issue. In these profiles, two distinct clusters of characteristics emerged. In the first were younger participants with less formal education and less experience with PER, and who self-reported as being less knowledgeable about PER in general. They were more likely than the others to be worried about PPRs’ professionalization, power dynamics between researchers and patients, exploitation of vulnerable persons, and paternalism. The other cluster consisted mainly of older participants with more formal education, more experience with PER, and more self-professed knowledge about PER. They were more likely to worry about the issues of patient remuneration, recognition of patient contributions, instrumentalizing funding, and confidentiality. This distinction is hardly surprising; it is to be expected that experienced ECRs would focus more on the applied and concrete aspects, while juniors with less practical experience would still be concentrating on theoretical issues.

**Table 1:** Ranking of the most pressing ethical issues

Rank	Ethical issue	Profile of panelists more likely to select the issue
1	Emergence of a class of professional patients (a reduced pool of patients with specific profiles which PER projects continually draw upon)	Younger, lower education level, student, less knowledgeable about PER, no experience with engaging PPRs
2	Patient remuneration	Older, higher education level, more knowledgeable about PER
3	Fair recognition and appreciation of patient expertise	No longer students, more knowledgeable about PER, experience with engaging PPRs
3	Using PER for the financial opportunities it presents to researchers without actually applying it once funding has been secured	No longer students
5	Power sharing between researchers and patients	Younger, lower education level, student, less knowledgeable about PER, no experience with engaging PPRs
5	Confidentiality	Higher education level, no experience with engaging PPRs
5	Exploitation of vulnerable persons	Student, less knowledgeable about PER
8	Paternalism and its off-shoots	Younger, lower education level, student
9	Educating patients about the world of research (structure, protocol format, validity criteria, etc.)	Older, higher education level, professional, more knowledgeable about PER, experience with engaging PPRs
10	Educating patients about research integrity	N/A (chosen by only one person)

The following sections present the three main ethical issues identified by panelists: PPRs’ professionalization, remuneration of patients, as well as authorship and fair recognition of patients’ contribution.

### PPRs’ professionalization

The most pressing issue, for the panel as a whole, was the emergence of a class of professional PPRs, that is, a small number of patients with specific and sought-after profiles who are continuously (re)engaged in PER projects. One panelist warned particularly against the emergence of professional PPRs, arguing it could be disastrous as, over time, these recruited patients come to resemble the researchers more than the patient community. Since professional PPRs would be screened according to specific inclusion criteria, the concern is that they would ultimately become less representative of the community of patients, being recruited primarily to satisfy researcher needs and preferences, which may not actually accommodate or welcome diversity.

This resonates with the concept of “proto-professionalism” suggested by de Swaan et al. [21, 22]. Caron-Flinterman et al. applied this concept in the context of PER with reference to patients’ internalization of (bio)medical scientific language and principles [23]. Patients who participate in the research process as experts and are in direct contact with the team almost inevitably undergo a process of professionalization, in the sense that, for instance, through reading scientific articles and discussing with professionals, patients internalize (bio)medical and professional knowledge, which becomes integrated into their experiential knowledge [24]. Consequently, according to Caron-Flinterman et al., “[p]roto-professionalism may lead to non-representation of the patient community and to the loss of ‘pure’ experiential knowledge” [24], which echoes panelists’ warnings about frequently re-engaging the same PPRs in research projects, a concern shared by Ives et al. [25].

Besides proto-professionalism, another concern raised by panelists was that professional PPRs might end up pursuing personal agendas, such as building up their research *curriculum vitae* or defending the interests of professional PPRs rather than of the entire patient community. From this may arise conflicts of interest between their career as PPRs—a personal source of self-esteem—and their role and duties of representing the patient’s perspective.

### Remunerating patients

The second most important issue was patient remuneration; in fact, panelists who self-identified as knowledgeable about PER considered it most pressing. Panelists lamented a general lack of funding for patient remuneration, fairly low success rates for PER in funding competitions, and lack of knowledge and guidance on what constitutes fair remuneration. Patients’ remuneration is an essential dimension because it has an impact on the feasibility of engaging patients in research, as panelists perceived that PPRs might be reluctant to invest in a project without proper compensation. Guidance is also needed on remuneration standards to fairly compensate PPRs and on how to engage PPRs ethically when resources are scarce. This tension was perceived as being more challenging for ECRs, since they may have more difficulty and fewer opportunities than their senior colleagues to secure the necessary funds to compensate PPRs properly for their time and investments; this is especially true for graduate students conducting research not funded by their supervisor.

What emerged from the results was the importance of the issue of inequity due to insufficient PPR compensation, when PPRs are the only members of the research team who are not (or only minimally) financially compensated. While guidelines do exist outlining compensation expectations for PPRs [26–28], it is unclear what should be done in the absence of sufficient funds and other resources. Some panelists therefore raised as an ethical dilemma the question of what to do in the absence of sufficient funds and how to balance their willingness to conduct a PER project—building on the strengths of PPRs’ input and the positive impacts of a PER approach—with an inability to adequately and fairly compensate PPRs.

### Authorship and fair recognition of patients’ contributions

The third most pressing issue pertains to recognizing patients’ expertise and contributions. This was discussed particularly with regard to patients’ eligibility for scientific authorship.

**Fig. 2:**
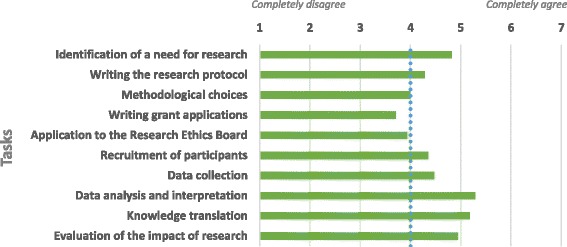
Tasks in which PPRs must participate to be considered co-authors

The rules regarding contributions required for authorship vary greatly among disciplines and research contexts [29–31]. Considering that patient involvement may differ from one project to another, panelists were asked what aspects of research PPRs had to be involved in to be considered co-authors. The issue of authorship was deemed important because directly underlying it are the crucial issues of fair recognition and involvement of PPRs, areas where panelists saw room for improvement. Figure 2 presents the tasks in which PPRs were expected to participate to be recognized as co-authors.

Panelists tended to agree that PPRs were mainly expected to be involved in the early (identification of a research need) and late (data analysis and interpretation, knowledge translation, evaluation of research impact) stages of a research project. This finding complements, while diverging slightly from, a literature review by Domecq et al., which concluded that researchers most commonly tend to involve patients in the beginning of a project (agenda setting, study design and procedures, and study recruitment) and in results implementation [32]. One difference was that ECRs in the present study were in agreement around involving PPRs in data analysis and interpretation (i.e., in the “late stage” of the execution phase of the research), whereas few studies documented this in Domecq et al.’s review.

## Discussion

**Fig. 3:**
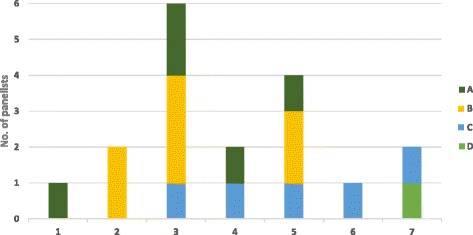
Self-perceived readiness to independently prepare and submit an ethics certificate application (research ethics board approval). 1. Completely disagree to 7. Completely agree. A. Becoming familiar with PER, B. Basic knowledge of PER, C. Advanced knowledge of PER, D. Mastery of PER

Beyond values and the most pressing ethical issues, ECRs’ ethical preparedness was, in itself, an important concern. When asked whether they felt equipped to independently prepare and submit a PER application to a research ethics board for an approval (ethics certificate), panelists were particularly divided (m = 3.94). Unsurprisingly, those who were experienced with and knowledgeable about PER felt much better prepared (see Fig. 3). This suggests that while it is necessary to support and help to prepare ECRs with meaningful and practical ethical training, ECRs concurrently develop their own ethical awareness and become better prepared to handle ethical challenges as they encounter them and develop practical experience.

Four panelists indicated the need for a concerted strategy on the part of funding agencies, research networks, researcher communities, and universities to support researchers in conducting PER and confronting ethical challenges. One panelist suggested that funding agencies put in place a compulsory online course for new investigators, with a certificate upon completion, to ensure they understand the ethical standards for conducting PER. To be effective and relevant, these initiatives should take into account the particular characteristics of ECRs. A national survey conducted in the US among 1479 ECRs and 1768 mid-career researchers examined associations between training and mentoring on the responsible conduct of research and reported ethically problematic behaviors that could compromise responsible research conduct [33]. Certain problematic behaviors raised in that study are of interest in relation to the ethical issues raised by the panelists; these involved: methods (e.g. knowingly using inadequate research designs or withholding important methodological details); outside influence (e.g. “modifying research directions or agendas to fit the priorities of funders”); inappropriate use of funds (e.g. using funds in a way that differed from the funded protocol); credit (e.g. inappropriately assigning authorship credit); policy (e.g. “ignoring major aspects of human-subjects requirements”), and cutting corners (e.g. skimping on important tasks in the haste to complete a project). Even though the study was not centered on PER, two important findings seem relevant to ECRs’ ethical preparedness. First, ECRs are more exposed to ethical training than are mid-career respondents, which may suggest it is propitious to act early in their career. Second, different forms of mentoring produce different results, and some types may have undesirable effects on ethical behaviors. Ethics mentoring (e.g. “discussions on ethical issues with instructors, mentors, or colleagues”) lowered the odds of problematic behavior in the areas of methods and cutting corners. Research mentoring (e.g. good research practices) was inversely related to problematic behavior in the areas of methods, use of funds, and cutting corners. Personal mentoring (e.g. ongoing interest and emotional support) lowered the odds of questionable behavior in the categories of methods and outside influence. In contrast, financial (e.g. guidance on writing grant requests and obtaining financial support) and survival (e.g. guidance on how to survive in academia) mentoring were associated with increased odds of reported problematic use of funds, and survival mentoring had a similar association with methods-related behaviors. These findings are instrumental to understanding the importance and impacts of mentoring, and to recognizing that some types of mentoring may exacerbate ethical issues. Thus, preparing the next generation of researchers calls for providing ethical supports (e.g. training and mentoring) that foster appropriate behavior (including compliance with research ethics boards’ expectations), while taking into consideration the practical and survival dimensions as strong determinants of researchers’ behavior.

### Tokenism

Over the Delphi rounds, one dimension that emerged in relation to all ethical issues raised by panelists was tokenism, or doing something merely for its symbolic appeal. This concern was in line with many studies concluding that tokenism is a predominant issue [11, 32, 34]. According to panelists, tokenism can take two forms, instrumentalizing the fund-seeking process and instrumentalizing patients.

#### Instrumentalizing funding

Panelists acknowledged that they might “consider modifying a research protocol to make it eligible for competitions restricted to PER projects” (m = 4.39). Interestingly, this evokes the highly-ranked (#2/10) ethical issue of instrumentalizing PER to obtain funding but then not genuinely conducting PER once funding is secured. This tension between the (non-negligible) probability of engaging in such practices (i.e., modifying a research protocol) and the underlying ethical issue might be explained by ECRs’ belief that integrating PPRs into their research projects represents a career asset/opportunity (m = 5.39). Panelists perceived adequate integration of patients and their perspectives into research as very difficult to achieve, due either to lack of resources and experience, or to the nature of the research conducted. This concern was shared by the panelists, the CIHR, and other researchers [11, 32, 35]. 

#### Instrumentalizing patients

Panelists were mostly concerned that, because PER practices can be challenging and demanding, researchers might end up making only tokenistic use of patients. In that scenario, patients would have a window-dressing role rather than an authentic place within research projects. Panelists’ concerns were congruent with Snape et al.’s warning of public involvement tokenism becoming a self-fulfilling prophecy [36], which in the PER context would mean that, as PER is difficult to conduct, employing tokenistic practices involving non-representative patients―or simply engaging patients suboptimally―undermines the very reason for involving PPRs and may lead to less impactful research. As reported by a researcher in Lough’s article, “one thing that pisses [patients] off more than not being involved is being involved and being ignored” [11].

**Fig. 4:**
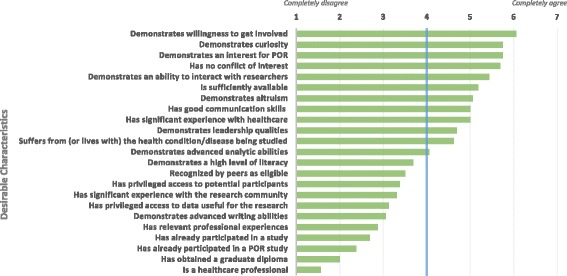
Desirable characteristics in PPR recruitment

In response to this predicament, when asked about the characteristics they would want to see in PPRs, the panelists’ own answers defused their concerns. Figure 4 presents the extent to which panelists valued the PPR characteristics they had identified in an earlier round.

The PPR characteristics most desired by the panelists were mainly related to willingness to contribute to research, interest in PER, curiosity, ability to interact with the research team, and absence of conflicts of interest. The least desired characteristics were having a graduate diploma and being a healthcare professional. Selecting patients based on these most desired characteristics is a step towards moving beyond surface-level patient engagement. Focusing on willingness to contribute as a core criterion for recruitment is, in fact, a good way to avoid tokenism.

According to Vat et al. [37], patients’ characteristics (such as desire to help, time availability) are only one factor that can potentially influence the recruitment and retention of patients in research; other factors include environments (e.g. social and organizational) conducive to meaningful integration, opportunities for contribution, as well as education and mentorship and various forms of support. Although those authors did not use the label ‘professional PPRs’ per se, their respondents described the representativeness issue qualitatively as the difficulty of recruiting a diversity of patients with varied profiles, while noting that members of the research team were not, for instance, held to the same standard of representativeness (e.g. an economist on the team was not assumed to be representative of all economists). Among our panelists, the characteristics desired seemed to be more of a moral and representational nature rather than being determined by feasibility and logistical imperatives.

### When ethical desiderata confront logistical imperatives

An important tension to consider from the panelists’ perspectives is the apparent clash between the ethical desiderata and the logistical imperatives of PER. While PER appears to be presented in the literature as an ethical imperative, it can be difficult to ensure PER feasibility without confronting the ethical pitfalls of tokenism. While access to additional financial resources and support can help relieve the instrumentalization of funding, patient instrumentalization is a much more complex issue. It may be partly defused by providing a suitable environment (social, organizational) for involving patients and enabling them to contribute wholly to research, and by recruiting PPRs who are more representative of the broader lay patient community and possess panelists’ ideal characteristics. However, the latter does not guarantee their genuine and efficient inclusion in a research team [37], and selecting for characteristics such as willingness, curiosity, interest in PER, and absence of conflicts of interest may not be achievable and might even lead to inefficient PER. Theoretically ideal PPRs may, in practice, and for a variety of logistical reasons, fail to integrate and to contribute effectively to a research project or to advise decision-makers. Thus, PPRs’ professionalization could potentially be inevitable, because integrating and effectively involving ‘naïve’ (i.e., inexperienced) patients in a research team may simply be more difficult, whereas recruiting certain types of PPRs with ‘effective’ characteristics and continuously re-engaging ‘experienced’ PPRs could render PER increasingly feasible. Conversely, recruiting only professional PPRs could potentially exclude patients from vulnerable populations, which would have significant ethical consequences on the representation and inclusion of marginalized communities.

This raises the question of whether preference should be given to PPRs with panelists’ ideal characteristics (albeit arguably more difficult to recruit and involve) or to professional PPRs (ready to join a team and having from the outset the skills to contribute to a project). While most PPRs will be positioned at different points along the spectrum rather than at the poles, any reflection on the desired involvement of patients in research will necessarily entail some degree of pragmatism as well as an assessment of the necessary conditions to foster the mobilization of the experiential knowledge from those living with a particular health condition. Professionalized or not, the objective of recruitment must certainly be that PPRs are not recruited to please researchers, but to lead to effective and authentic PER.

## Limitations

This study was limited to 16 participants, all from Quebec. Although the sample was small and geographically limited, the high participation rate, the panelists’ varied profiles, and the use of three rounds allowed for in-depth analysis. It is important to note that there was no statistical hypothesis testing, and what has been presented seemingly as quantitative results are trends within a limited group of ECRs. This is the first study of its kind, offering an initial enunciation and discussion of ethical issues associated with PER, an emerging research approach that should be further explored in other contexts and jurisdictions.

## Conclusions

If PER is itself an ethical imperative, it is vital to establish clear ethical standards and to train and support the PER community to identify and resolve ethical issues. Our study panelists called for clearer ethical guidelines, especially from those involved in the PER ecosystem (funding agencies, universities, research networks, methodological clusters, etc.). Such resources might include advice on best ethical practices for patient inclusion in research teams, and guidelines on accountability, research integrity, and avoiding instrumentalization of patients and funding. These guidelines would benefit all actors involved in PER.

A common theme raised by the panelists (especially those less experienced with PER) was that, despite their overall readiness to conduct PER, they did not feel adequately prepared to address these ethical issues. This points to a possible gap in and need for a support system to prepare ECRs for conducting PER (including engaging patients in all stages of research) and managing the associated ethical issues. Our panelists expected research institutions (funding agencies, universities, research networks, etc.) to establish guidelines to equip and support ECRs in the ethical conduct of PER.

The concerns about instrumentalization challenge current practices and raise the question of whether, for researchers, patient engagement is a means or an end (or both). The limited resources available to ECRs push them too often, despite their good intentions, to a paradox. On the one hand, PER is increasingly valued and encouraged within the research community, due to its inclusive practices, valuing of patients’ experiential knowledge, and power sharing. On the other hand, conducting PER is logistically challenging, which can lead to only surface-level patient engagement and raises a wide range of ethical issues regarding PER’s underlying values, responsible conduct, social utility, and authorship. As such, logistical considerations often trump ethical ones, while PER is often lauded for its moral implications. Acting on the ethical dimensions and responsible conduct of research also requires financial and logistical support.

To our knowledge, this is the first empirical study examining ethical aspects of PER. Further research is required on the issues identified in this study. Specifically, four questions merit further exploration: 1) how ethical involvement of PPRs is defined; 2) what constitutes fair recognition of PPRs’ knowledge and contribution; 3) whether PPR professionalization is desirable, and if so, what guidelines are needed to ensure the ethical conduct of these new professionals in research; and 4) how tokenism in PER can be avoided (and whether professionalization fosters or prevents tokenism). In considering these questions, the responsibilities of the actors involved in PER (e.g. funding agencies, universities, research networks, research teams) to support research communities in addressing ethical issues should also be defined. This is crucial to ensuring PER actually involves genuine and efficient engagement of patients in research. Much work is needed to establish ethical standards for and responsible conduct in engaging patients in research, which, if not done, can undermine the credibility and feasibility of the entire PER enterprise.

## Abbreviations

CIHR: Canadian Institutes of Health Research
ECR: Early-career researcher
m: Mean
PER: Patient engagement in research
PPR: Patient-partners in research
